# Probabilistic graphical models relate immune status with response to neoadjuvant chemotherapy in breast cancer

**DOI:** 10.18632/oncotarget.25496

**Published:** 2018-06-12

**Authors:** Andrea Zapater-Moros, Angelo Gámez-Pozo, Guillermo Prado-Vázquez, Lucía Trilla-Fuertes, Jorge M. Arevalillo, Mariana Díaz-Almirón, Hilario Navarro, Paloma Maín, Jaime Feliú, Pilar Zamora, Enrique Espinosa, Juan Ángel Fresno Vara

**Affiliations:** ^1^ Molecular Oncology & Pathology Laboratory, Institute of Medical and Molecular Genetics-INGEMM, La Paz University Hospital-IdiPAZ, Madrid, Spain; ^2^ Biomedica Molecular Medicine SL, Madrid, Spain; ^3^ Operational Research and Numerical Analysis, National Distance Education University, Madrid, Spain; ^4^ Biostatistics Unit, La Paz University Hospital-IdiPAZ, Madrid, Spain; ^5^ Department of Statistics and Operations Research, Faculty of Mathematics, Complutense University of Madrid, Madrid, Spain; ^6^ Medical Oncology Service, La Paz University Hospital-IdiPAZ, Madrid, Spain; ^7^ CIBERONC, Madrid, Spain

**Keywords:** breast cancer, neoadjuvant chemotherapy, molecular subtypes, probabilistic graphical models, immune status, Immunology

## Abstract

Breast cancer is the most frequent tumor in women and its incidence is increasing. Neoadjuvant chemotherapy has become standard of care as a complement to surgery in locally advanced or poor-prognosis early stage disease. The achievement of a complete response to neoadjuvant chemotherapy correlates with prognosis but it is not possible to predict who will obtain an excellent response. The molecular analysis of the tumor offers a unique opportunity to unveil predictive factors. In this work, gene expression profiling in 279 tumor samples from patients receiving neoadjuvant chemotherapy was performed and probabilistic graphical models were used. This approach enables addressing biological and clinical questions from a Systems Biology perspective, allowing to deal with large gene expression data and their interactions. Tumors presenting complete response to neoadjuvant chemotherapy had a higher activity of immune related functions compared to resistant tumors. Similarly, samples from complete responders presented higher expression ​​of lymphocyte cell lineage markers, immune-activating and immune-suppressive markers, which may correlate with tumor infiltration by lymphocytes (TILs). These results suggest that the patient’s immune system plays a key role in tumor response to neoadjuvant treatment. However, future studies with larger cohorts are necessary to validate these hypotheses.

## INTRODUCTION

Breast cancer is the most common neoplasm and the fifth cause of cancer-associated death among women [1]. Estrogen receptor (ER), progesterone receptor (PR), and human epidermal growth factor receptor 2 (HER2) provide a system of classification and clinical diagnosis. Seventy percent of the tumors are hormonal receptor positive, and HER2 overexpression is observed in 15% of cases. ER+ and PR+ tumors respond to endocrine therapy, whereas tumors overexpressing HER2 respond to targeted therapies such as trastuzumab [2, 3]. Tumors negative for ER, PR and HER2 are known as Triple Negative Breast Cancer (TNBC) and do not respond to the aforementioned therapies.

A molecular classification of breast cancer defined four intrinsic subtypes [4]. Luminal A disease, which accounts for 67% of all tumors, shows high expression of genes related to hormone receptors and low expression of genes related to cell proliferation. Luminal B, HER2-enriched and Basal-like subtypes have a more aggressive phenotype [5] [6, 7].

Neoadjuvant chemotherapy has been increasingly administered to reduce the size of primary tumor, thus increasing the likelihood of breast conservation and enhancing survival [8]. Currently, there is no clinically useful molecular predictor of response to cytotoxic drugs in the neoadjuvant setting. Clinical parameters or the expression of single molecular markers (ie, Bcl-2, p53, MDR-1, and so on) show weak association with response and are not regimen-specific. Molecular subtyping may offer some help, as Luminal B and Basal-like tumors respond better than Luminal A tumors [9], but this is not accurate enough to make clinical decisions. As a consequence, many patients suffer the toxicity of useless neoadjuvant chemotherapy.

This study has been carried out using probabilistic graphical models, providing insights into the molecular biology of tumor response, allowing its use as a predictive model for response. These statistically inferred networks provide a deeper level of biological understanding in two main directions: giving support to previously identified biological observations, and giving new insights regarding novel biological interactions. Moreover, the transcriptional network approach has proven to be useful to unveil transcriptional regulation in breast cancer [10, 11]. The objective of this study was to evaluate differences in gene expression patterns of breast cancer tumors from patients who had undergone neoadjuvant chemotherapy through a Systems Biology perspective.

## RESULTS

### Patient’s characteristics

279 patients with histologically-confirmed primary non-metastatic breast adenocarcinoma from phase II trial (NCT00455533) [12] were included. They all had untreated tumors of at least 2 cm in size (T2-3, N0-3) regardless of hormone receptor or HER2 expression status. Clinical data were obtained from phase II trial (NCT00455533). Patient’s clinical characteristics are provided in Table 1. On the basis of ER, PR and HER2 status, 111 tumors patients (39.78%) were ER+ or PR+ and Her2- (ER+ for now on), 28 (10.04%) were HER2+ and 140 (50.18%) were classified as TNBC. Patients received sequential neoadjuvant therapy starting with 4 cycles of doxorubicin/cyclophosphamide (AC), followed by 1:1 randomization to either ixabepilone or paclitaxel. All patients underwent definitive breast surgery 4 to 6 weeks after the last dose of ixabepilone or paclitaxel, consisting of either a lumpectomy with axillary dissection or modified radical mastectomy. Regarding pathological response, 40 (14.34%) patients achieved a complete response (CR), 161 (57.71%) achieved a partial response (PR), 64 (22.94%) had stable disease (SD) and 5 (1.79%) had progressive disease (PD).

**Table 1:** Patient’s clinical characteristics

Characteristic	Patients (*n*)	Patients (%)	Characteristics	Patients (*n*)	Patients (%)
Age			Pathological response		
**Mean age**	48.63		CR	40	14.34%
**≤50**	166	59.50%	PR	161	57.71%
**>50**	113	40.50%	PD	5	1.79%
**Tumor size (T)**			SD	64	22.94%
**< 2 cm**	3	1.08%	Unassigned	9	3.23%
**2 - 5 cm**	174	62.37%	**ER status**		
**> 5 cm**	99	35.48%	ER+	108	38.71%
**Unassigned**	3	1.08%	ER-	171	61.29%
**Nodal classification (N)**			**PR status**		
**N0**	122	41.40%	PR+	99	35.48%
**N1**	136	46.10%	PR-	179	64.16%
**N2**	30	10.20%	Unknown	1	0.36%
**N3**	7	2.40%	**HER2 status**		
**Neoadjuvant treatment**			HER2+	28	10.04%
**Ixabepilone**	138	49.46%	HER2-	251	89.96%

### Molecular stratification of tumors

Molecular subtypes were defined by PAM50 assignment [13]. Of the initial 279 patients, 116 (41.58%) patients were classified as Basal-like subtype, 15 patients (5.38%) as HER2+, 66 (23.66%) as Luminal A, 62 (22.22%) as Luminal B, and 15 (5.38%) as Normal-like. Five patients could not be assigned due to Spearmn’s rank correlation were not statistically significant for neither of the molecular subgroup centroids. A sub-classification of TNBCs was performed based on Lehmann’s classification as previously described [14]; 25 (8.96%) TNBC tumors were Basal-like 1, 83 (29.75%) Basal-like 2 subgroup, 6 (2.15%) Luminal Androgen Receptor, and 26 (9.32%) Mesenchymal.

### Breast cancer systems biology

**Figure 1:**
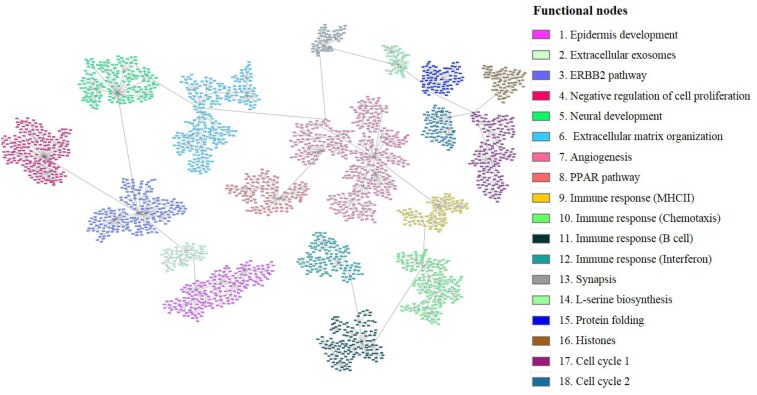
Breast cancer network Probabilistic graphical model from 279 tumors gene expression data divided in eighteen functional nodes harboring one or two predominant biological functions. Each node (box) represents one gene and each grey line (edges) connects genes with correlated expression.

Gene expression data from all tumor samples were used to build a probabilistic graphical model, with no other *a priori* information. The resulting graph was divided in eighteen branches (functional nodes) and a main function was assigned to each node by gene ontology analysis. The structure of the probabilistic graphical model clearly reflected different biological functions (Figure 1) Functional node activities were then calculated as previously showed [10, 15].

### Functional structure of response to neoadjuvant chemotherapy

**Figure 2:**
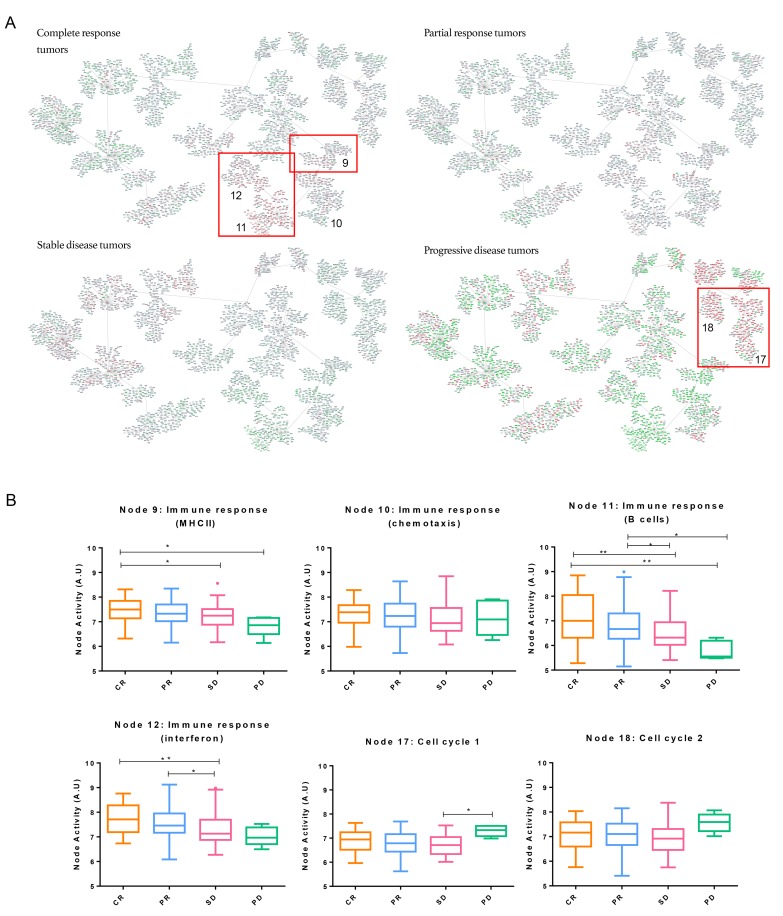
Breast cancer network by pathological response groups **A.** Detail of nodes with the highest activity in each of the subgroups. Genes with an expression below 0 were represented in green; genes with an expression around 0 were represented in grey and genes with an expression above zero were represented in red. **B.** Functional node activities differences between pathological response groups: Box-and-whisker plots are Tukey boxplots. All *p*-values were two-sided and *p* < 0.05 was considered statistically significant. *P*-value < 0.05 (*); *p*-value < 0.01(**). A.U: arbitrary units.

Patients were classified according to pathological response regardless of their tumor molecular subtype to study the response to neoadjuvant chemotherapy. Significant differences between functional node activities were observed in “Immune response (MHCII)” (node 9), “Immune response (B cell)” (node 11) and “Immune response (Interferon)” (node 12) nodes, in which, tumors attaining a complete response had higher activation (Figure 2). Blown up pictures of the genes in the red boxes are provided in Supplementary Figures 1-5. A progressive decrease in the activity of immune functional nodes was seen depending on the response, being higher in tumors obtaining a CR and absent in those having a progression. Additionally, the relationship of immune nodes activities with the pathological response was evaluated using an ordinal logistic regression analysis. This analysis revealed that an increment of one unit in node 9, 11 and 12 activities increased the probability of a favorable response 1.739, 1.435 and 1.629 times respectively. By contrast, one unit increase in the activity of node 10 increased 0.519 times the probability of having an unfavorable response. On the other hand, PD tumors showed higher functional activity in “Cell cycle 1” (node 17) and “Cell cycle 2” (node18), followed by CR tumors.

### Functional characterization of molecular subtypes

**Figure 3:**
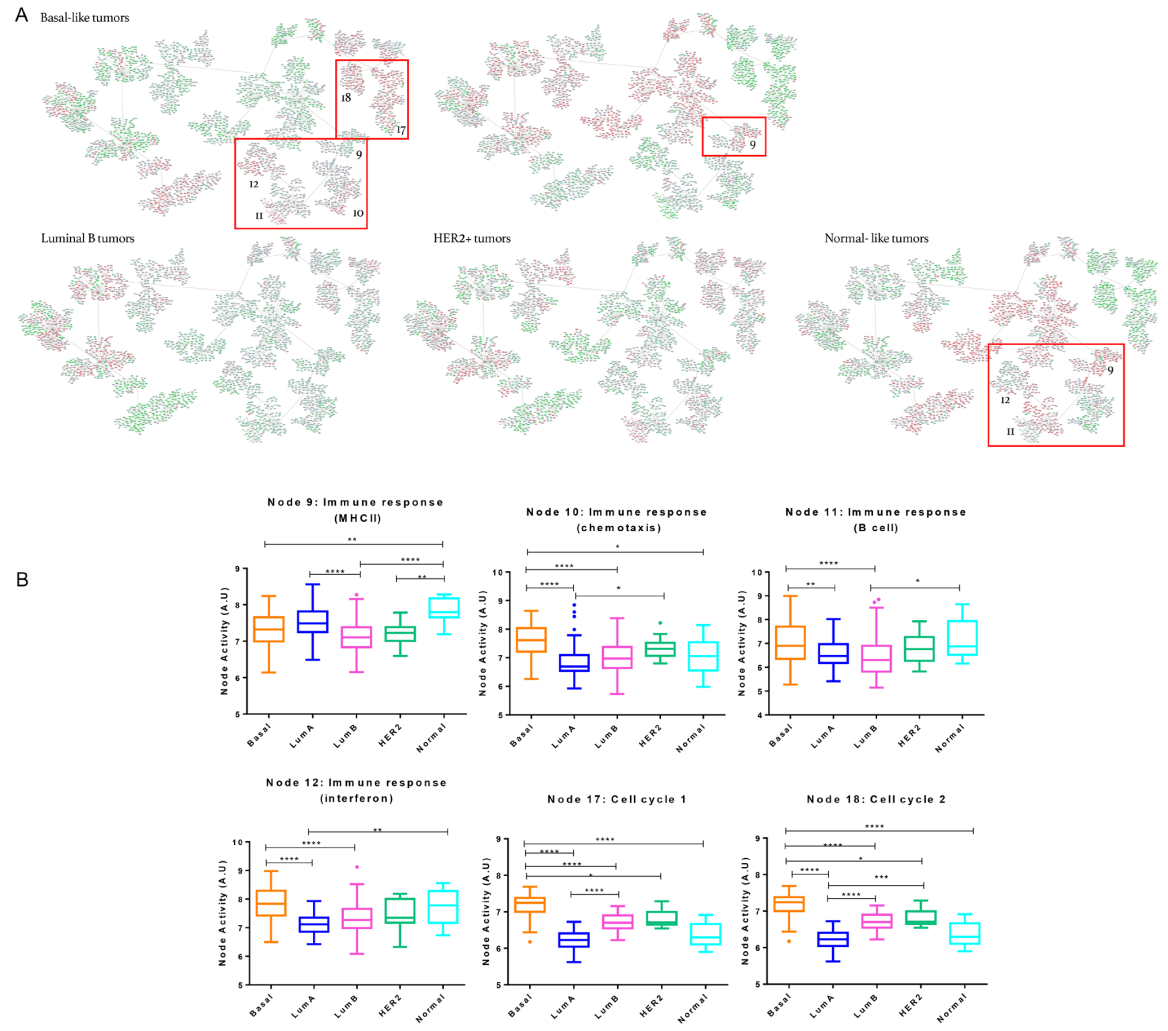
Breast cancer network by breast cancer molecular subtypes **A.** Detail of nodes with the highest activity in each of the subgroups. Genes with an expression below 0 were represented in green; genes with an expression around 0 were represented in grey and genes with an expression above zero were represented in red. **B.** Functional node activities differences between molecular subtypes: Box-and-whisker plots are Tukey boxplots. All *p*-values were two-sided and *p* < 0.05 was considered statistically significant. *P*-value < 0.05 (*); *p*-value < 0.01(**); *P*-value< 0.001 (***); *P*-value <0.0001 (****). A.U: arbitrary units.

Patients in the network were further classified according to their molecular subtype (Basal-like, Luminal A, Luminal B, HER2+ and Normal-like). Basal-like tumors were also classified according to Lehmann’s subtypes. “Immune response (MHCII)” (node 9) node activity was higher in Luminal A and Normal-like subtypes while Basal-like tumors showed higher functional node activity in “Immune response (chemotaxis)” (node 10), “Immune response (B cell)” (node 11) and “Immune response (Interferon)” (node 12), as well as in “Cell cycle 1 and 2” (nodes 17 and 18) nodes (Figure 3).

Concerning TNBCs sub-classification, BL2 subtype showed a higher functional activity in “Immune response” (nodes 9, 10, 11 and 12) nodes whereas it was observed higher “Cell cycle 1 and 2” (nodes 17 and 18) nodes activities in BL1 tumors.

In order to evaluate the functional implications between molecular subtypes and response to neoadjuvant therapy, data from patients of the same molecular subtype were mean centred and analysed independently. Luminal A group included no PD, whereas only one PD was found in Luminal B group, and was excluded from this analysis. Normal-like and HER2+ tumors were insufficient to perform subsequent analyses.

Concerning Luminal A and Luminal B subtypes, “Immune response (MHCII)” (node 9), “Immune response (chemotaxis)” (node 10), “Immune response (B cell)” (node 11) and “Immune response (Interferon)” (node 12) functional nodes activities were higher in tumors attaining a CR, although differences were not statistically significant. As in the case of Basal-like tumors, a progressive decrease of activity in these nodes was observed from CR to SD.

In Basal-like subtype, tumors attaining a CR showed significant differences in “Immune response (B cell)” (node 11) and “Immune response (Interferon)” (node 12) nodes activities while “Immune response (MHCII)” (node 9) node activity was higher in tumors showing a PR. PD tumors showed a higher functional node activity in Cell cycle 1” (node 17) and Cell cycle 2” (node 18). Regarding TNBC, in the BL1 subtype, the activity of nodes “Immune response (B cell)” (node 11) and “Immune response (Interferon)” (node 12) was higher in CR than in PR. “Immune response (MHCII)” (9), “Immune response (chemotaxis)” (node 10) node activities also were higher in CR tumors but without statistics differences. In the BL2 subtype, CR tumors showed a significant higher activity in “Immune response (MHCII)” (node 9) and “Immune response (B cell)” (node 11). However, SD tumors showed a higher activity in “Immune response (chemotaxis)” (node 10). In Mesenchymal subtype, CR tumors showed higher activity in “Immune response (MHCII)” (node 9) and “Immune response (B cell)” (node 11) nodes but without being statistically significant.

Three separate probabilistic graphical models were built for Basal-like, Luminal-A and Luminal-B subtypes. As in the global network, tumors experiencing a CR had an increased activity of immune response-related nodes, although differences were not statistically significant.

### Immunological markers

**Figure 4:**
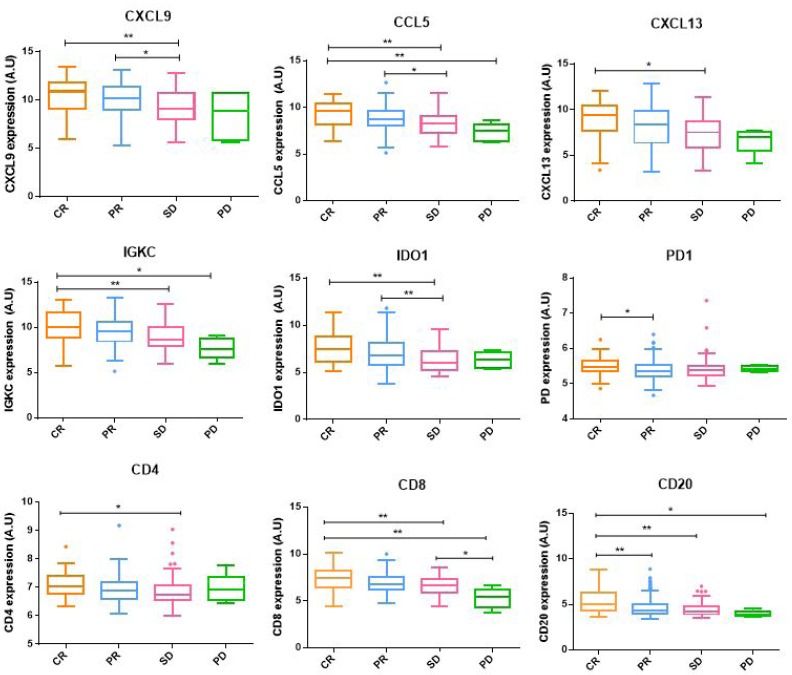
Immunological markers expression Immune-activating, immunosuppressive and cell lineage markers gene expression between pathological response groups. Box-and-whisker plots are Tukey boxplots. All *p*-values were two-sided and *p* < 0.05 was considered statistically significant. *P*-value < 0.05 (*); *p*-value < 0.01(**); *p*-value < 0.001 (***); *p*-value <0.0001 (****). A.U: arbitrary units.

Markers of tumor-infiltrating lymphocytes were analysed to further characterize response according to the immune status. For that, patients were separated according to their pathological response and marker gene expression levels were compared between groups. Tumors obtaining a CR showed significantly higher expression levels of cell lineage markers (CD4, CD8 and CD20) as well as immune-activating (IGKC, CXCL9, CCL5, CXCL13) and immunosuppressive markers (IDO1, PD1) compared to the rest of tumors (Figure 4).

## DISCUSSION

In this work, a gene expression-based probabilistic graphical model analysis of breast cancer showed that immune functional nodes were related to pathological response to neoadjuvant chemotherapy. This correlation did not depend on the molecular subtype, indicating that a Systems Biology approach complements knowledge obtained from other research methods. Non-directed probabilistic graphical models allow managing large gene datasets and underscoring lots of gene interactions, many of which have not been previously described.

The activity of immune nodes was higher in tumors attaining a CR and decreased with the intensity of response. Tumors progressing on chemotherapy also showed increased activity in the nodes “Cell division 1” (node 17) and “Cell division 2” (node 18). These results suggest that the patient’s immune system plays a crucial role in the response to neoadjuvant chemotherapy. Previous studies suggest that conventional therapies are effective in patients exhibiting some degree of immune activation in the tumor [16], supporting our findings. Chemotherapy may mediate the “immunogenic” death of tumor cells, thus facilitating an immune response against the disease [17].

As expected, all tumors attaining a CR- regardless of molecular subtype- showed significantly higher levels of cell lineage markers (CD4, CD8 and CD20) as well as immune-activating (IGKC, CXCL9, CCL5, CXCL13) and immunosuppressive markers (IDO1, PD1), suggesting a greater infiltration of immune cells High tumor-infiltrating lymphocytes (TILs) levels have been associated with increased CR rates in ER+ HER2+/- tumors [18] and also in TNBC [19]. However, high levels of PD-1+ TILs or Foxp3+ TILs have been related with poor prognosis [18]. Therefore, immune cell subpopulation profiles could help to predict response to neoadjuvant chemotherapy.

Basal-like and HER2+ subgroups have been associated with highest CR rates as opposed to Luminal and Normal-like tumors. However, the genes that were associated with CR in Basal-like subgroup were not associated with CR in the HER2+ subgroup, suggesting that different sets of genes are associated with CR in the different molecular subtypes [20, 21]. In the present cohort, Basal-like tumors had the highest CR rate, as expected. However, the CR rate was poor in HER2+ tumors, probably because these patients did not receive anti-HER2 targeted therapy. On the other hand, BL1 tumors achieved a CR more commonly than other TNBC subtypes, as previously described [22]. Although node “Immune response (MHCII)” (node 9) had higher activity in Luminal A and Normal subtypes, the remaining nodes related to immune response showed increased activity in Basal-like tumors. This agrees with the fact that, in general, there are far fewer TILs in luminal disease than in HER2 or TNBC subtypes [23]. In fact, even though increased TILs concentrations are associated with increased frequency of response to neoadjuvant chemotherapy in all breast cancer subtypes, there is a different effect of TILs on survival in TNBC and luminal breast cancer. Increased TILs concentrations are associated with longer survival in TNBC and HER2+ disease, but not in luminal-HER2-negative tumours [24], suggesting again a differences in the biology of the immunological infiltrate across molecular subtypes.

One possible explanation of the higher “Immune response (MHCII)” (node 9) activity in Luminal A subtype could be the contribution of different immune cell types. Most types of immune cells were increased in TNBC compared with luminal-HER2- negative breast cancer. In TNBC, the presence of many immune cell subtypes, including B cells, T cells, and macrophages, were linked to improved survival [24]. By contrast, in luminal-HER2-negative breast cancer, the presence of T cells was not prognostic for survival and the only cell types linked to improved prognosis were B and myeloid dendritic cells [24], which are MHCII presenting cells. Taking this into account, it would be interesting to perform further studies about MHCII presenting cells infiltration in luminal subtypes.

On the other hand, Basal-like tumors also had the highest activity in the node “Cell cycle” (nodes 17 and 18), which is in accordance with the fact proliferation renders tumor cells more sensitive to chemotherapy [6].

The neoadjuvant setting is appealing in the field of drug development because it allows early evaluation of efficacy. However, not all patients benefit from this approach, so markers predicting response to neoadjuvant chemotherapy are clearly necessary, as neoadjuvant therapy may have some drawbacks, such as promoting metastasis in some cases [25]. Our results suggest that immune activation in the tumor may identify responders. Although validation is needed, the use of these markers can help to determine the future use of neoadjuvant chemotherapy in breast cancer.

## MATERIALS AND METHODS

### Patient’s and samples origin and characteristics

A breast cancer tumor dataset, including gene expression and clinical data, was obtained from the Gene Expression Omnibus (GSE41998) and from a phase II trial (NCT00455533). Gene expression profiling was measured using an Affymetrix GeneChip, normalized and log2 transformed. Surgical specimens were evaluated by a pathologist at each study site. The pathological response was evaluated as the primary endpoint. A pathological complete response (CR) was defined by no histologic evidence of residual invasive adenocarcinoma in the breast and axillary lymph nodes, with or without the presence of ductal carcinoma in situ [12]. Responses were categorized as complete, partial, stabilization or progression.

### Gene expression data preprocessing

The PAM50 method was used as previously described to assign a molecular subtype to each sample [13]. Lehmann subtypes for TNBC were assigned in two steps [14]. First, samples were assigned to Lehmann’s seven subtypes using centroids constructed from 77 previously assigned tumors in GSE31519 dataset. Then, the IM and MSL groups were redefined as previously described [14].

### Probabilistic graphical models construction

A functional structure was developed using undirected probabilistic graphical models (PGMs) as previously described [10]. Briefly, PGMs compatible with high-dimensionality were chosen. The result is an undirected graphical model with local minimum Bayesian Information Criterion. DAVID 6.8 was used to assign a biological function to each node in the networks, using “homo sapiens” as background list and selecting only GOTERM-FAT and Biocarta and KEGG pathways categories. Functional activity of each node was calculated by the mean expression of the genes in each node. To visualize node activities, data from all tumors used to construct the network were mean centred prior to its inclusion into the network.

### Statistics and software suites

Differences between groups were assessed using Kruskal-Wallis test, Mann-Whitney test and Dunn`s multiple comparisons test using GraphPad Prism 6. Box-and-whisker plots are Tukey boxplots. All p-values were two-sided and p<0.05 was considered statistically significant. Ordinal logistic regression analysis was performed in SAS using logistic procedure. Network analyses were performed in MeV and Cytoscape 3.2.1 software suites.

## SUPPLEMENTARY MATERIALS FIGURES



## Abbreviations

AC: doxorubicin/cyclophosphamide
BL1: Basal-like 1
BL2: Basal-like 2
CR: complete response
ER: Estrogen receptor
HER2: Human epidermal growth factor receptor 2
IM: immunomodulatory
MSL: mesenchymal stem-like
PD: progressive disease
PGMs: probabilistic graphical models
PR: Partial response
PR: Progesterone receptor
SD: stable disease
TNBC: Triple Negative Breast Cancer
