# Supplementary material for: Probabilistic graphical models relate immune status with response to neoadjuvant chemotherapy in breast cancer
Source: Oncotarget. 2018 Jun 12;9(45):27586–94. doi: 10.18632/oncotarget.25496 (PMC6021258; doi:10.18632/oncotarget.25496)
Supplement: Supplementary file 1 [file oncotarget-09-27586-s001.pdf]

Probabilistic graphical models relate immune status with response to neoadjuvant chemotherapy in breast cancer

SUPPLEMENTARY MATERIALS

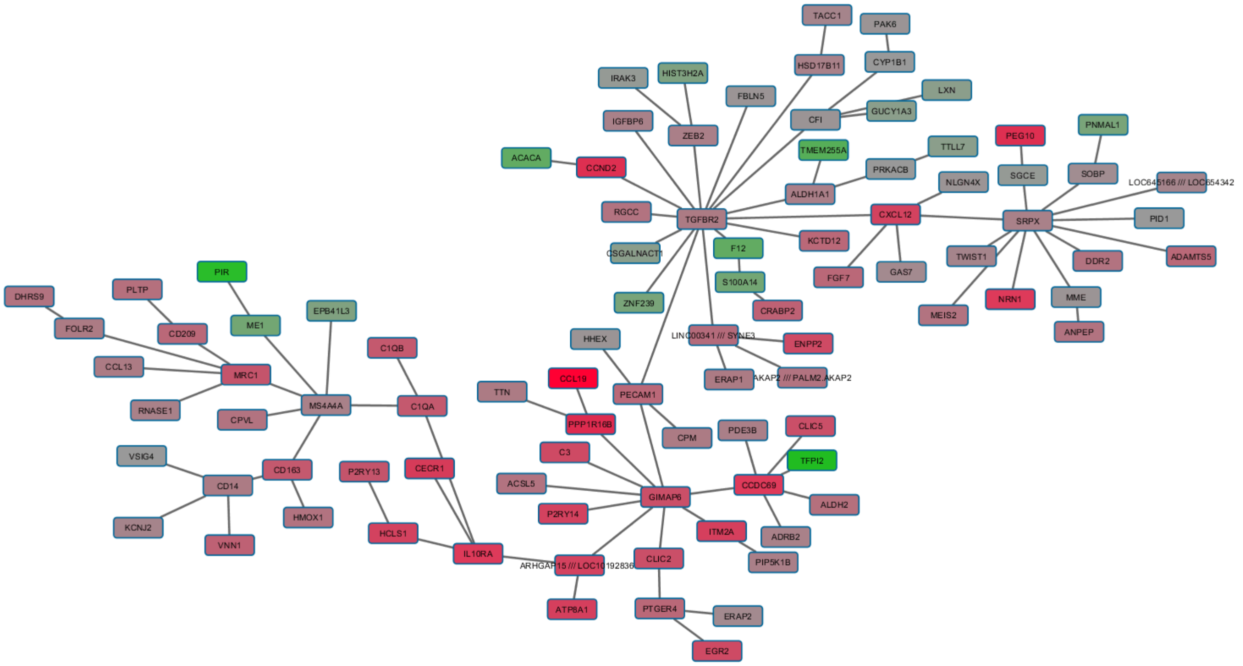

Supplementary Figure 1: Detail of "Immune response (MHCII)" (node 9) in Complete Response tumors. Genes with an expression below 0 were represented in green; genes with an expression around 0 were represented in grey and genes with an expression above zero were represented in red.

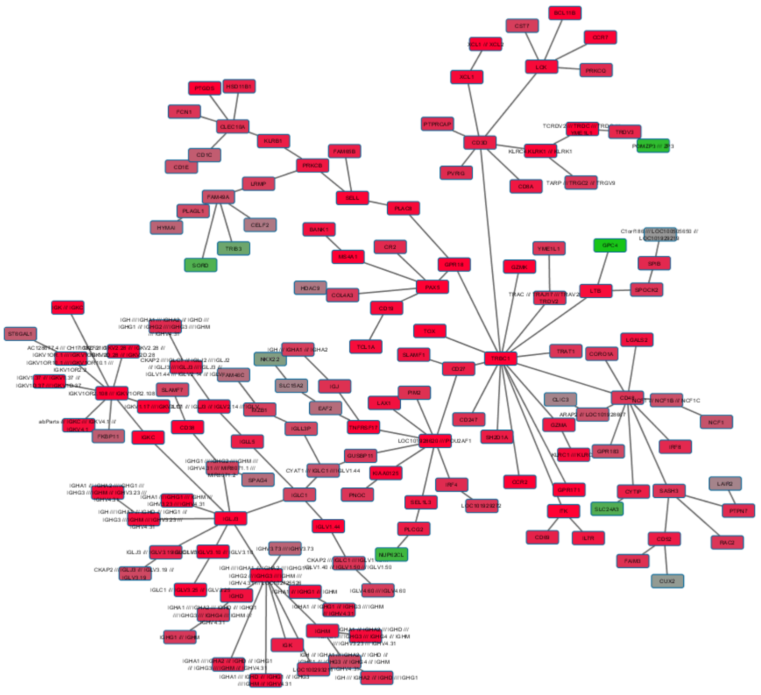

Supplementary Figure 2: Detail of "Immune response (B cell)" (node 11) in Complete Response tumors. Genes with an expression below 0 were represented in green; genes with an expression around 0 were represented in grey and genes with an expression above zero were represented in red.

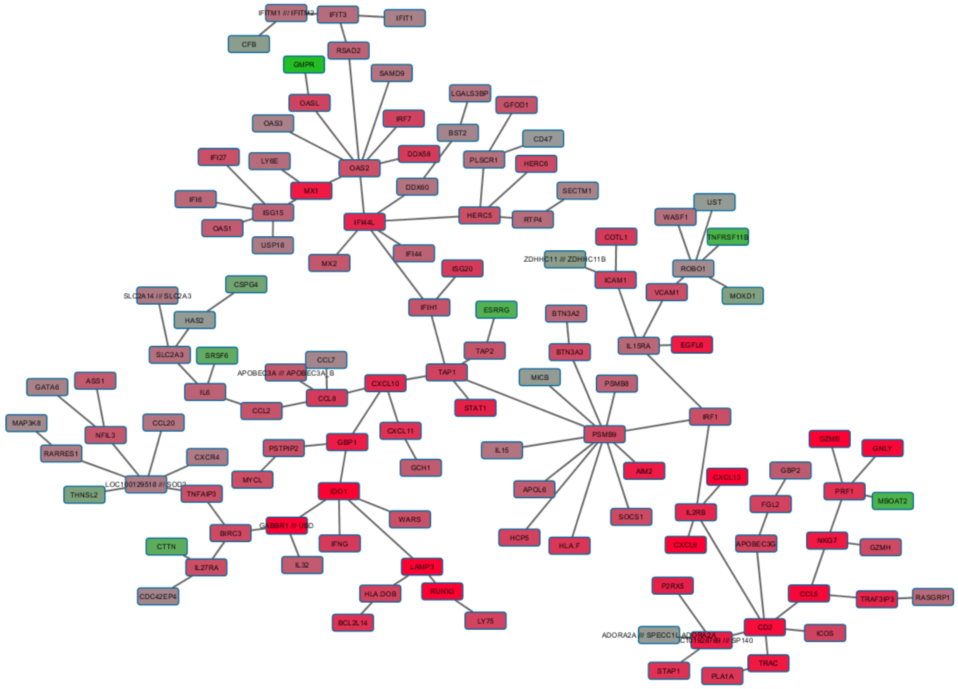

Supplementary Figure 3: Detail of "Immune response (Interferon)" (node 12) in Complete Response tumors. Genes with an expression below 0 were represented in green; genes with an expression around 0 were represented in grey and genes with an expression above zero were represented in red.

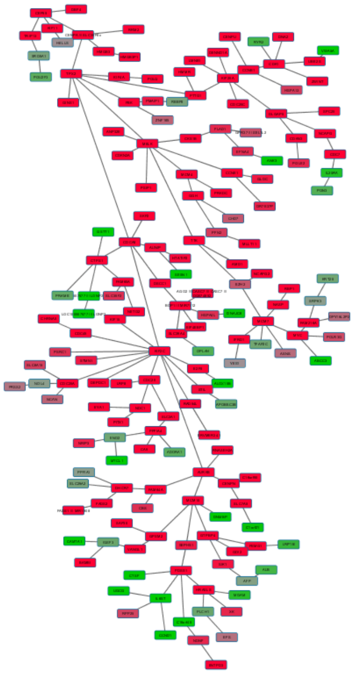

Supplementary Figure 4: Detail of "Cell Cycle 1" (node 17) in Progressive Disease tumors. Genes with an expression below 0 were represented in green; genes with an expression around 0 were represented in grey and genes with an expression above zero were represented in red.

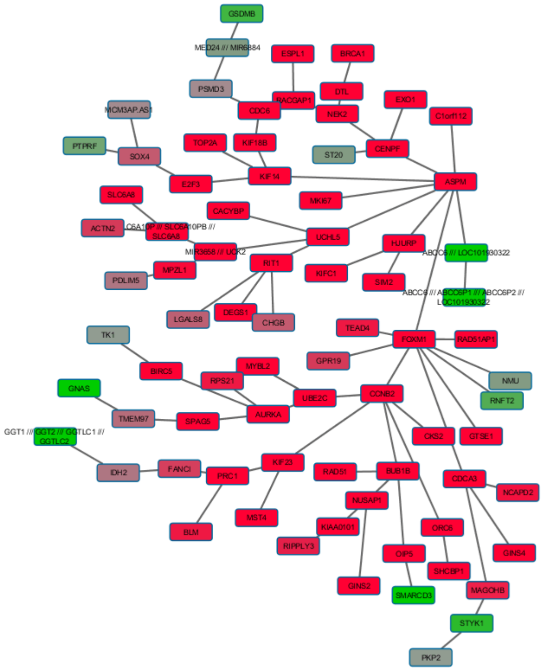

Supplementary Figure 5: Detail of "Cell Cycle 2 (node 18) in Progressive Disease tumors. Genes with an expression below 0 were represented in green; genes with an expression around 0 were represented in grey and genes with an expression above zero were represented in red.
